# Therapeutic hypothermia augments the restorative effects of PKC-β and Nox2 inhibition on an in vitro model of human blood–brain barrier

**DOI:** 10.1007/s11011-021-00810-8

**Published:** 2021-08-16

**Authors:** Rais Reskiawan A. Kadir, Mansour Alwjwaj, Zoe McCarthy, Ulvi Bayraktutan

**Affiliations:** Academic Unit of Mental Health and Clinical Neuroscience, School of Medicine, The University of Nottingham, Hucknall Road, Nottingham, NG5 1PB UK

**Keywords:** Hyperglycaemia, Stroke, Therapeutic hypothermia, Protein kinase C, Oxidative stress, Blood–brain barrier

## Abstract

To investigate whether therapeutic hypothermia augments the restorative impact of protein kinase C-β (PKC-β) and Nox2 inhibition on an in vitro model of human blood–brain barrier (BBB). Cells cultured in normoglycaemic (5.5 mM) or hyperglycaemic (25 mM, 6 to 120 h) conditions were treated with therapeutic hypothermia (35 °C) in the absence or presence of a PKC-β inhibitor (LY333531, 0.05 μM) or a Nox2 inhibitor (gp91ds-tat, 50 μM). BBB was established by co-culture of human brain microvascular endothelial cells (HBMECs) with astrocytes (HAs) and pericytes. BBB integrity and function were assessed via transendothelial electrical resistance (TEER) and paracellular flux of sodium fluorescein (NaF, 376 Da). Nox activity (lucigenin assay), superoxide anion production (cytochrome-*C* reduction assay), cellular proliferative capacity (wound scratch assay) and actin cytoskeletal formation (rhodamine-phalloidin staining) were assessed both in HBMECs and HAs using the specific methodologies indicated in brackets. Therapeutic hypothermia augmented the protective effects of PKC-β or Nox2 inhibition on BBB integrity and function in experimental setting of hyperglycaemia, as evidenced by increases in TEER and concomitant decreases in paracellular flux of NaF. The combinatory approaches were more effective in repairing physical damage exerted on HBMEC and HA monolayers by wound scratch and in decreasing Nox activity and superoxide anion production compared to sole treatment regimen with either agent. Similarly, the combinatory approaches were more effective in suppressing actin stress fibre formation and maintaining normal cytoskeletal structure. Therapeutic hypothermia augments the cerebral barrier-restorative capacity of agents specifically targeting PKC-β or Nox2 pathways.

## Introduction

Ischaemic stroke stemming from an interference with blood supply leading to or within the brain continues to be one of the main causes of mortality and morbidity worldwide. Disruption of the blood–brain barrier (BBB) and ensuing vasogenic oedema constitute the main causes of neurological impairment during the acute phase of ischaemic strokes (Alwjwaj et al. 2021; Jiang et al. 2018). Prolonged exposure to hyperglycaemia in patients with diabetes mellitus (DM) amplifies the extent of stroke-mediated BBB disruption through thickening of the capillary basement membrane and increases in oxidative stress, neuronal and endothelial cell apoptosis (Venkat et al. 2017). Unsurprisingly, ischaemic stroke patients with DM suffer worse outcomes than those without (Thorén et al. 2017; Yu et al. 2016). Hyperglycaemia also negates the beneficial effect of the recombinant tissue plasminogen activator (rtPA), the only approved pharmaco-therapeutic agent, and is coupled to higher rates of intracranial haemorrhage (Masrur et al. 2015). Since less than 5% of patients with ischaemic stroke receive rtPA due to a narrow therapeutic window (first 4.5 h of stroke onset), search for an alternative therapeutic regimen continues (Kadir et al. 2020; Kadir and Bayraktutan 2019). Although mechanical thrombectomy somewhat addresses the issue pertaining to limited therapeutic time window, it is only effective in ischaemic stroke stemming from large vessel occlusion and requires advanced facilities and technical resources for application (Turc et al. 2019). Hence, exploration of novel therapeutic agents and approaches to treat patients continues to be a pressing need in stroke medicine.

Therapeutic hypothermia, defined by a core body temperature ≤ 35 °C, is routinely used to minimise deleterious effects in global hypoxia-mediated cerebral injury in comatose patients suffering cardiac arrest and in neonates with hypoxic-ischaemic encephalopathy (Bernard et al. 2002; Shankaran et al. 2005). The application of therapeutic hypothermia with rtPA or mechanical thrombectomy appears to be safe and feasible for patients with ischaemic stroke and is associated with reduced infarct size and neurological deficits in animal model of ischaemic stroke (Chen et al. 2016; Piironen et al. 2014; van der Worp et al. 2007). However, the effect of therapeutic hypothermia in experimental and clinical settings associated with hyperglycaemia remains unknown.

Oxidative stress, characterised by excessive availability of reactive oxygen species (ROS), in particular superoxide anion, has been shown to account for much of the hyperglycaemia-mediated appearance of endothelial dysfunction and ensuing vascular complications. Indeed, endothelial dysfunction represents a key early and potentially reversible event in diabetic ischaemic stroke to which activation of NADPH oxidase (Nox) and PKC-β appear to contribute the most (Bayraktutan 2002; Shao and Bayraktutan 2013),. Amongst all Nox isoforms, Nox2 is more closely associated with the cerebral injury (Kahles and Brandes 2012). Hence, it is no surprise that the inhibition of either PKC-β or Nox2 activity shows a protective effect on BBB, most likely due to inactivation of Rho kinase, attenuation of oxidative stress and reorganisation of actin cytoskeleton formation (Kahles et al. 2007; Shao and Bayraktutan 2013).

In light of the available data, the current study investigates whether strategies combining therapeutic hypothermia with agents targeting either PKC-β or Nox2 may better protect the integrity and function of an in vitro model of human BBB against hyperglycaemic insult. Furthermore, the present study also investigates how these combinatory approaches compare against the single treatment regimens for wound repair, oxidative stress regulation and actin cytoskeletal formation using the two main components of BBB, human brain microvascular endothelial cells (HBMECs) and astrocytes.

## Materials and methods

### Cell culture

HBMECs, human astrocytes (HAs) and human pericytes (HPs) were purchased from TCS CellWorks Ltd. (Buckingham, UK) and cultured in respective specialised media (Sciencell, Caltag Systems, Buckingham, UK) in a humidified atmosphere under normal conditions (75% N_2_, 20% O_2_ and 5% CO_2_). In related studies, cells were cultured to about 60% confluence and were subjected to normoglycemia (5.5 mM D-glucose) or hyperglycaemia (25 mM D-glucose) in the presence or absence of hypothermia (35ºC), PKC-β inhibitor (LY333531, 0.05 μM, Sigma) or Nox2 inhibitor (gp91ds-tat, 50 μM, SynPeptide, Shanghai, China). The concentration of the inhibitors used in the current study were selected according to the previous studies (Rey et al. 2001; Shao and Bayraktutan 2013, 2014; Zinkevich et al. 2017). To investigate the effect of combinatory approaches, LY333531 or gp91ds-tat were added to culture media and exposed to therapeutic hypothermia. The culture media was changed every other day during extended exposure to hyperglycaemia (6–120 h). Cell culture under normoglycemic and normothermic conditions served as controls. Considering that various therapeutic agents such as Rho kinase inhibitor (Gibson et al. 2014), anti-oxidant compounds (catalase, vitamin C and E) (Allen and Bayraktutan 2009), NADPH oxidase inhibitor (Abdullah and Bayraktutan 2014), anti-TNF-α (Abdullah et al. 2015), and calcium inhibitor (Rakkar and Bayraktutan 2016) protected BBB integrity and function against ischaemic/reperfusion, hyperglycaemic, or TNF-α injury in our previous studies and those studies utilised similar experimental settings, such positive control was not utilised in the present study. Moreover, given that exposure to therapeutic hypothermia in clinical settings equally affects both damaged and normal cells, we have investigated whether and how hypothermia modulates the parameters studied in the current study in cells maintained under normoglycaemic conditions (Bobi et al. 2020). However, PKC-β or Nox2 pathways are targeted only in pathological conditions such as hyperglycaemic injury, their effects in physiological settings have not been probed. Furthermore, as increases in osmolality, attained by the addition of D-mannitol, did not affect BBB integrity and function as well as PKC-β and Nox signalling pathways in any way compared to normoglycaemic controls, the effect of osmolality has not been reassessed in this study (Shao and Bayraktutan 2014; Srivastava et al. 2013).

### Establishment of an in vitro model of human blood–brain barrier

Since astrocyte end feet cover the entire endothelial layer and the direct contact between astrocytes and endothelial cells in the presence of pericytes produce the highest electrical resistance compared to non-contact or monolayer models, the triple culture model consisting of HBMECs, HAs and HPs was used in the present study (Allen and Bayraktutan 2009; Shao and Bayraktutan 2013). In brief, approximately 7.5 × 10^4^ HAs were seeded on the basolateral side of polyester Transwell inserts (0.4 µm pore size, 12 mm diameter polyester membrane, High Wycombe, UK). Following overnight adherence, the inserts were inverted the correct way and placed into 12-well dishes containing fresh medium to grow to about 80% confluence. HBMEC (~ 5 × 10^4^ cells) were then added to the inner part of the insert and both cell layers were left to grow to full confluence. To set up the triple-culture model, these Transwell inserts were transferred to 12-well plate containing confluent pericytes.

### Assessment of BBB characteristics

The integrity and function of the BBB were assessed as before by measurements of transendothelial electrical resistance (TEER, World Precision Instruments, Hertfordshire, UK) and paracellular flux of low molecular weight permeability marker, sodium fluorescein (NaF, 376 Da), respectively (Rakkar and Bayraktutan 2016). After exposure to their respective treatments, TEER across cellular barrier was measured using STX electrodes and an EVOM resistance meter. Two independent readings were taken and the measurements of blank filter were subtracted from inserts seeded with cells. Values are shown as Ωcm^2^ based on surface area of insert membrane. To measure NaF flux, inserts were washed twice with Hank’s Balanced Salt Solution (HBSS) and subsequently transferred to fresh 12-well plates containing 2 mL of HBSS. NaF (500 μL, 10 μg/mL) was carefully added to the luminal chamber and samples were taken in triplicate from both the luminal and abluminal chambers after incubation for 60 min at 37 °C. The average concentration of dye in each chamber was determined by measuring the fluorescence (excitation 485 nm and emission 520 nm) of samples with FLOUstar Omega Plate Reader (BMG Labtech Ltd., UK). Flux was expressed by NaF clear volume from the luminal chamber and calculated by using the following formula [(reading from abluminal chamber × 500) / reading from luminal chamber].

### Wound scratch assay

HBMEC and astrocytes were seeded in 6-well plates (1.5 × 10^5^ cells/well) and grown to full confluence before scratching the monolayers with a p1000 micropipette tip to induce a wound. The cells were then washed with PBS to remove the debris. Considering higher proliferative rates of HBMECs compared to HAs, pictures of wound closure were taken 24 and 48 h after scratch for HBMECs and HAs, respectively*.* The wound closure rates were quantified as the percentage of difference in scratch area at the beginning (immediately after scratch) and at the indicated time points using ImageJ software (version 1.52 k, NIH, Maryland, USA). Given the difficulties associated with the visualisation of particular cell layers in the triple culture system, the proliferative capacity both HBMECs and HAs in the present study was assessed using HBMEC and HA monolayers subjected to wound scratch (Abdulkadir et al. 2020).

### Measurement of Nox activity and superoxide anion generation

Nox activity was measured with the lucigenin chemiluminescence assay as previously described (Rakkar and Bayraktutan 2016). The HBMECs or HAs homogenates (~ 50 μg) were incubated at 37 °C in assay buffer containing potassium phosphate buffer (300 mM, pH 7.0, Sigma), ethylene glycol tetraacetic acid (50 mM, Sigma), sucrose (1 M, Sigma) and lucigenin (200 μM, Sigma). To eliminate the contributions of other ROS-generating enzymes to overall superoxide anion generation, the specific inhibitors for nitric oxide synthase (NG-nitro-L-arginine methyl ester, 10 mM, Sigma), mitochondrial respiratory chain complex 1 (rotenone, 10 mM, Sigma), xanthine oxidase (allopurinol, 10 mM, Sigma) and cyclooxygenase (indomethacin, 10 mM, Sigma) were also added to assay buffer. NADPH (100 μmol/L, Calbiochem, UK) was added to initiate the reaction after 15 min. The reaction was monitored every minute for 2 h and the rate of reaction was calculated using a luminometer (FLUOstar Omega, BMG Labtech, Aylesbury, UK).

The levels of superoxide anion were detected by cytochrome-*C* reduction assay (Rakkar and Bayraktutan 2016). In brief, equal amounts of homogenate (100 μg) were incubated with assay buffer containing HEPES (1 M, Calbiochem, UK), ethylene glycol tetraacetic acid (50 mM, Sigma), sucrose (1 M, Sigma), mannitol (1 M, Sigma) and cytochrome *C* (800 μM, Sigma), for 1 h at 37 °C. Superoxide anion generation was measured as the reduction of cytochrome *C* and monitored with the change in absorbance at 550 nm using a plate reader (FLUOstar Omega, BMG Labtech, Aylesbury, UK).

### Immunocytochemistry

HBMECs and HAs were grown to about 80% confluence on coverslip. The cells were fixed and permeabilised with 4% paraformaldehyde/PBS for 20 min and with 0.1% Triton X-100/PBS for 15 min, respectively*.* The cells then were stained with 1 × rhodamine phalloidin for 30 min (Abcam, Cambridge, UK) in the dark room and the coverslips subsequently were mounted on glass slides. Each experiment was performed in triplicate and the representative images obtained from five random fields on each coverslip were analysed by two different investigators using fluorescence microscopy (Zeiss Axio Observer, Carl Zeiss Ltd, Cambridge, UK). The length of stress fibres formed was measured using ImageJ software (version 1.52 k, NIH, Maryland, USA). Due to the difficulties pertaining to effective visualisation of stress fibre formation in different cell layers of a triple culture model, F-actin staining were performed using the HBMEC and HA monolayers (Abdulkadir et al. 2020).

### Statistical analysis

Data are presented as the mean ± SEM from at least three independent experiments. Statistical analyses were performed using GraphPad Prism 8.0 statistical software package (GraphPad Software Inc). Data were analysed by one-way ANOVA followed by a Tukey post hoc analysis. *P* < 0.05 was considered as significant.

## Results

### Therapeutic hypothermia augments the barrier-restorative effects of PKC-β or Nox2 inhibition

**Fig. 1:**
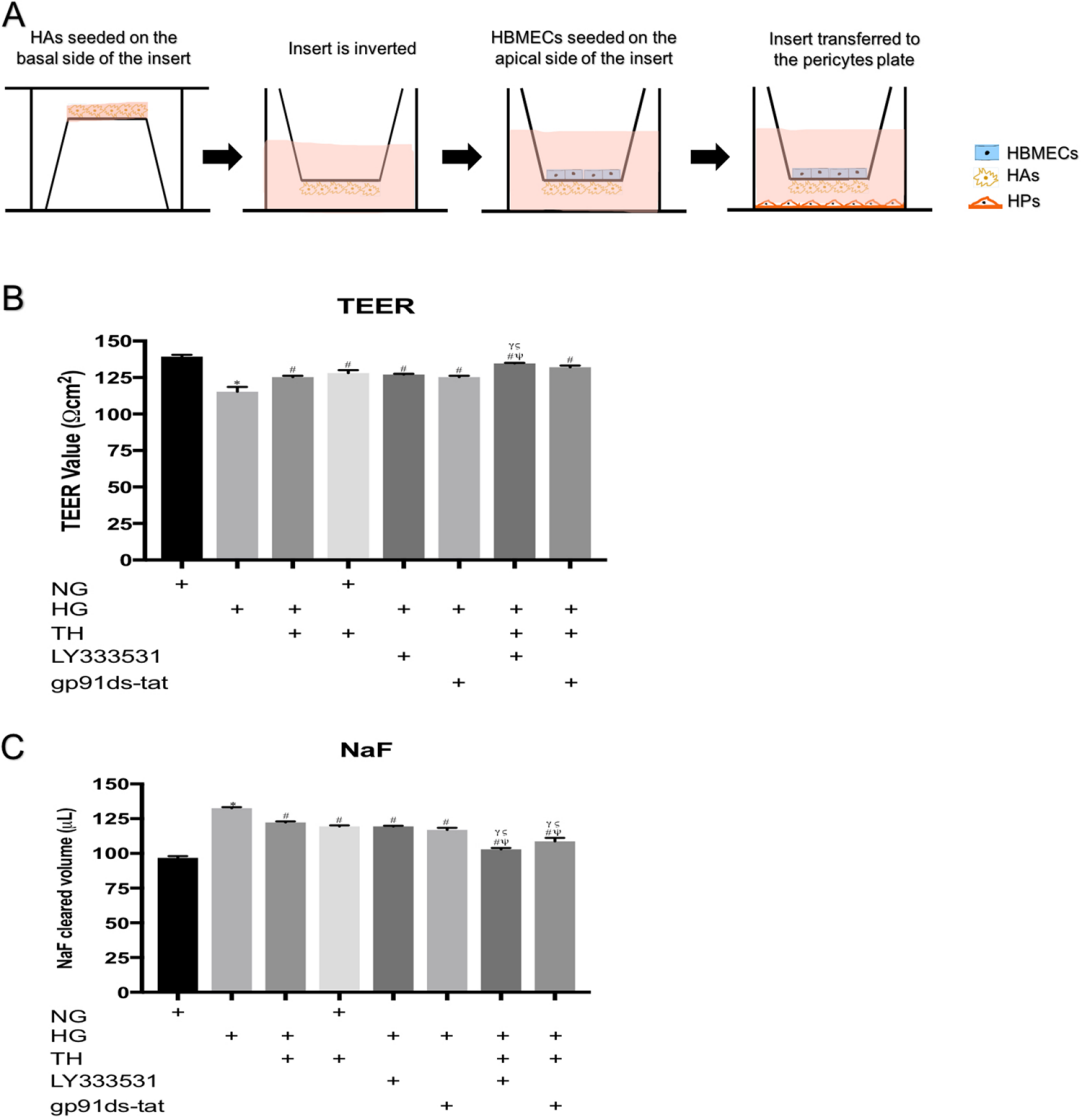
Schematic diagram summarising the establishment of an in vitro model of human BBB (**A**). The Transwell insert were inverted and human astrocytes (HAs) were seeded on the basal side of the insert. Once the cells were attached, the inserts were returned to their original orientation. Human brain microvascular endothelial cells (HBMEC) were seeded on the apical side of the insert once the HAs reached 90% confluence. The inserts were then transferred to a 12-well plate containing confluent human pericytes. The measurement of BBB integrity and function by TEER **B** and NaF flux **C**, respectively. BBB were exposed to 120 h hyperglycaemia and treated with therapeutic hypothermia alone or together with LY333531 and gp91ds-tat. While exposure to hyperglycaemia significantly compromised BBB integrity and function, treatment with single therapies i.e. therapeutic hypothermia, PKC-β, or Nox2 inhibitor attenuated the impact of HG on BBB integrity and function. The combinatory approaches showed better protection on BBB compared to any single treatment. Interestingly, exposure to therapeutic hypothermia in normoglycaemic settings disrupted BBB integrity and function, albeit the degree of impairment was substantially lesser than those induced by hyperglycaemia Data are expressed as mean ± s.e.m. from three different experiments. *P < 0.05 versus normoglycaemia (NG), ^#^*P* < 0.05 versus HG, ^Ψ^*P* < 0.05 versus HG + TH, ^γ^*P* < 0.05 versus HG + LY333531, and ^ς^*P* < 0.05 versus gp91ds-tat. NG, normoglycaemia; HG, hyperglycaemia; TH, therapeutic hypothermia

Exposure of the triple culture model of human BBB to hyperglycaemia significantly impaired BBB integrity and function, as observed by significant decreases in TEER and concurrent increases in paracellular flux of NaF across the barrier, respectively. Interestingly, exposure to therapeutic hypothermia (35 °C) alone also compromised the integrity and function of the BBB in normoglycaemic settings, albeit the magnitudes of impairment were significantly lesser than those induced by hyperglycaemia. While the inhibition of PKC-β or Nox2 improved BBB integrity and function in a similar manner to therapeutic hypothermia in hyperglycaemic settings, the combination of therapeutic hypothermia with either inhibitor almost completely neutralised the deleterious effect of hyperglycaemia on barrier integrity or function, proving a synergism in effect (Fig. 1).

### Therapeutic hypothermia accelerates the speed of wound repair induced by inhibition of PKC-β or Nox2

**Fig. 2:**
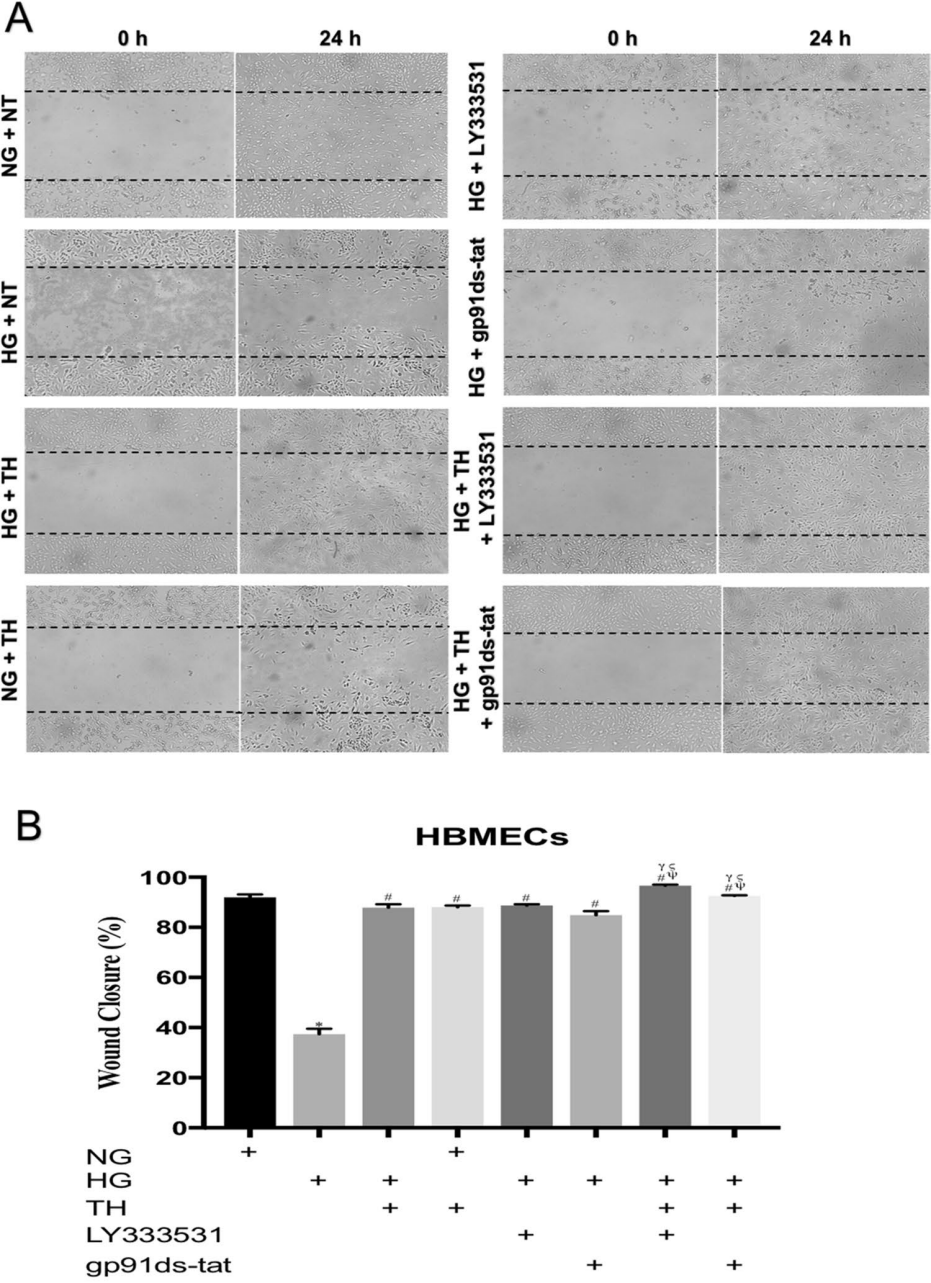
The effects of single or combinatory treatments with therapeutic hypothermia, LY333531 and gp91ds-tat on human brain microvascular endothelial cells (HBMEC) wound closure. While exposure to hyperglycaemia dramatically supressed the speed of HBMEC wound closure, the combination of therapeutic hypothermia with LY333531 or gp91ds-tat substantially accelerated HBMEC wound closure compared to all single treatment options (**A**). Moreover, the exposure of HBMECs to therapeutic hypothermia under normal conditions did not alter the wound reparative capacity of this particular cells. Graph showing the wound closure speed across all experimental groups (**B**). Data are expressed as mean ± s.e.m. from three different experiments. ^*^P < 0.05 versus normoglycaemia (NG), ^#^*P* < 0.05 versus HG, ^Ψ^*P* < 0.05 versus HG + TH, ^γ^*P* < 0.05 versus HG + LY333531, and ^ς^*P* < 0.05 versus gp91ds-tat. NG, normoglycaemia; HG, hyperglycaemia; TH, therapeutic hypothermia

**Fig. 3:**
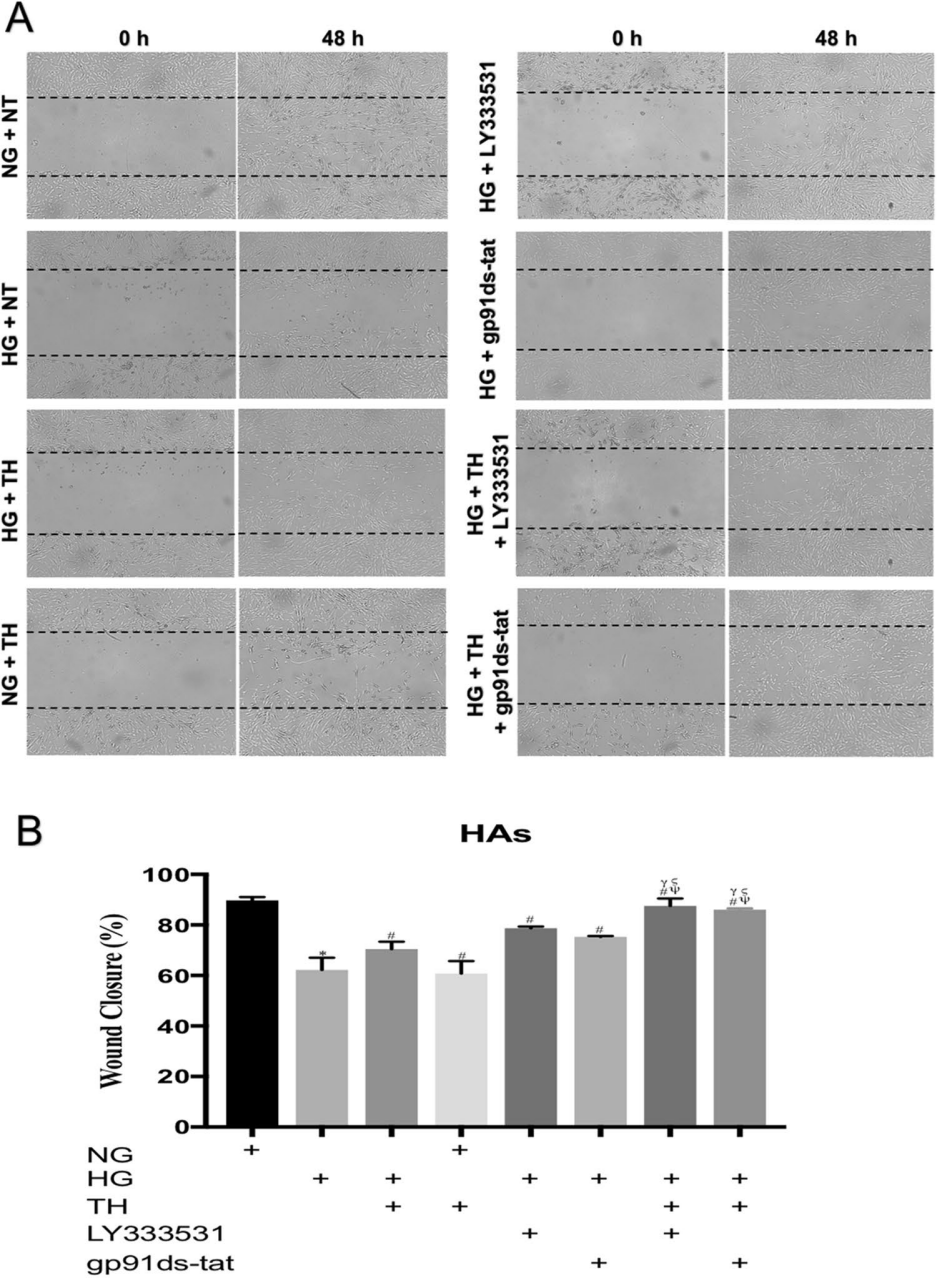
The effects of single or combinatory treatments with therapeutic hypothermia, LY333531 and gp91ds-tat on human astrocytes (HAs) wound closure. While hyperglycaemia dramatically impaired the speed of HA wound repair. The combination of therapeutic hypothermia with LY333531 or gp91ds-tat accelerated the rate of wound healing compared to any single therapeutic option. Co-exposure of HAs to therapeutic hypothermia and normoglycameia reduced the level HA wound repair to the levels observed in hyperglycaemic settings (**A**). Graph showing the wound closure speed across all experimental groups (**B**). Data are expressed as mean ± s.e.m. from three different experiments. ^*^P < 0.05 versus normoglycaemia (NG), ^#^*P* < 0.05 versus HG, ^Ψ^*P* < 0.05 versus HG + TH, ^γ^*P* < 0.05 versus HG + LY333531, and ^ς^*P* < 0.05 versus gp91ds-tat. NG, normoglycaemia; HG, hyperglycaemia; TH, therapeutic hypothermia

To ascertain how combining therapeutic hypothermia with an inhibitor for PKC-β signalling pathway or Nox2 enzyme affects the proliferation and migration of main cellular components of the BBB, a wound was generated on HBMEC and HA monolayers by scratch. The application of therapeutic hypothermia, PKC-β (LY333531) or Nox2 (gp91ds-tat) inhibitor alone repaired the physical damage introduced on HBMEC and HA monolayers in hyperglycaemic settings. Combination of therapeutic hypothermia with LY333531 or gp91ds-tat accelerated the reparative effect of both inhibitors compared to all single treatment options. Interestingly, therapeutic hypothermia adversely affected the proliferation rates of HBMECs and HAs under normoglyacemic conditions (Figs. 2 and 3).

### Therapeutic hypothermia potentiates the suppressive effects of PKC-β or Nox2 inhibition on oxidative stress

**Fig. 4:**
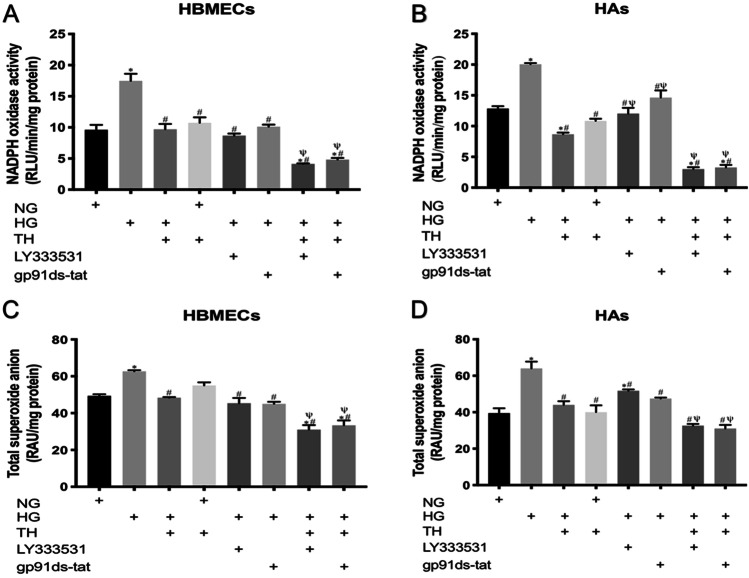
The effects of single or combinatory treatments with therapeutic hypothermia, LY333531 and gp91ds-tat on Nox2 activity and superoxide anion release in human brain microvascular endothelial cells (HBMEC) and astrocytes (HAs). Exposure of either cell line to hyperglycaemia led to significant increases in oxidase activity and superoxide anion generation. While treatment with single therapies effectively inhibited both parameters, co-application of therapeutic hypothermia with LY333531 or gp91ds-tat appeared to be more effective in reducing Nox2 activity **A** and **B** and superoxide anion release **C** and **D** in both HBMEC and HA subjected to hyperglycaemic insult for 120 h. Data are expressed as mean ± SEM from three different experiments. ^*^P < 0.05 versus normoglycaemia (NG), ^#^*P* < 0.05 versus HG, ^Ψ^*P* < 0.05 versus HG + TH, ^γ^*P* < 0.05 versus HG + LY333531, and ^ς^*P* < 0.05 versus gp91ds-tat. NG, normoglycaemia; HG, hyperglycaemia; TH, therapeutic hypothermia

Hyperglycaemia increased the level of superoxide anion in both HBMEC and HA through an increase in Nox activity. Therapeutic hypothermia as well as inhibition of PKC-β and Nox-2 by LY333531 and gp91ds-tat, respectively negated these increases in both cell lines. Combination of therapeutic hypothermia with either inhibitor decreased superoxide anion production and Nox activity below the levels seen in control cells, maintained in normoglycaemic and normothermic conditions. In contrast, exposure of normoglycaemic cells to therapeutic hypothermia did not alter the basal levels of superoxide anion production and Nox activity in either cell line (Fig. 4).

### Therapeutic hypothermia augments the protective effects of PKC-β or Nox2 inhibition on cytoskeleton

**Fig. 5:**
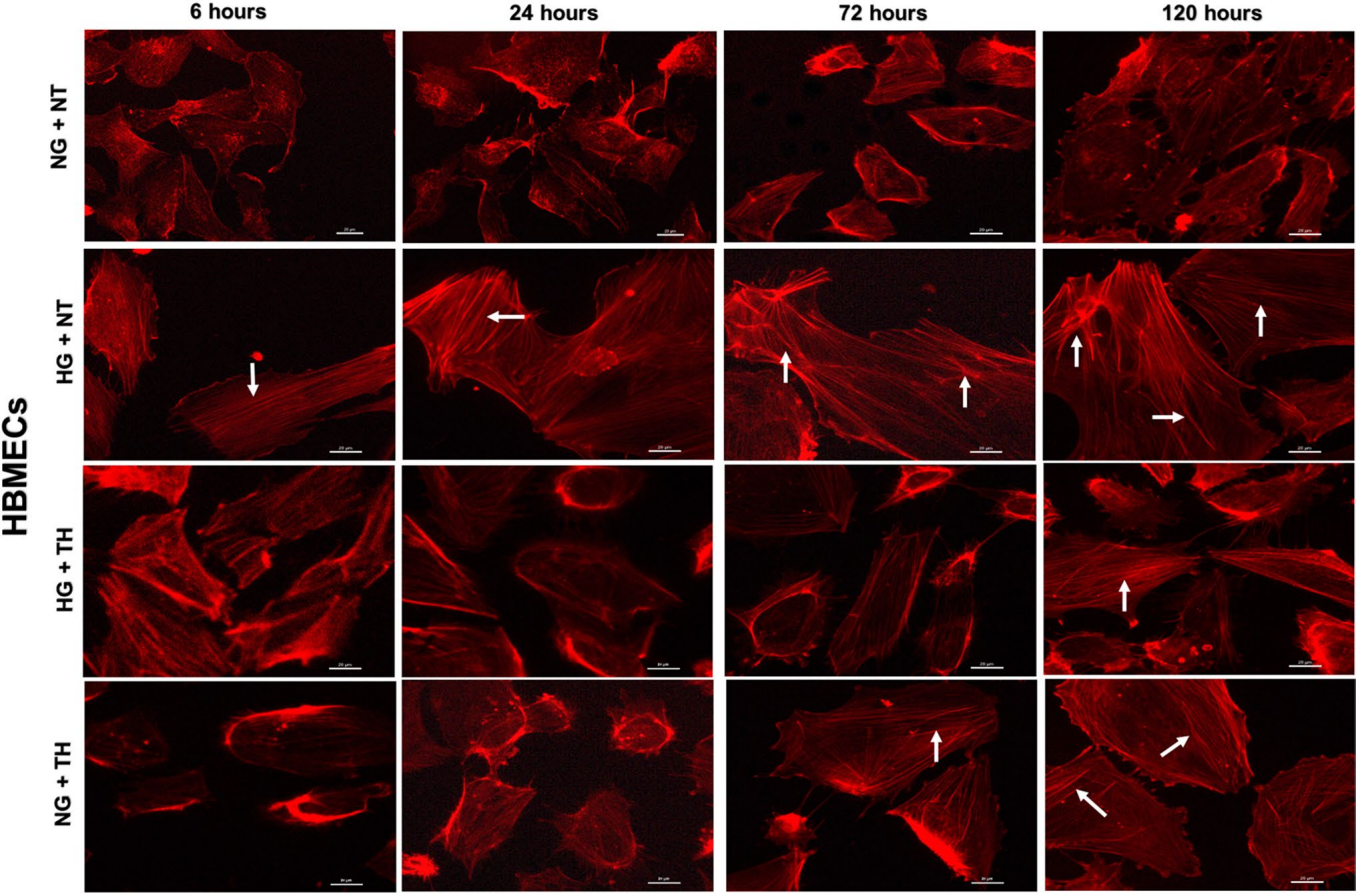
Representative images of actin cytoskeleton in human brain microvascular endothelial cells (HBMEC) cultured under normoglycaemic and hyperglycaemic (6–120 h) conditions in the absence or presence of therapeutic hypothermia. Treatment of normoglycaemic cells with therapeutic hypothermia or exposure to hyperglycaemic insult led to prominent actin stress fibre formation (indicated by white arrows) in HBMEC in a time-dependent manner. Therapeutic hypothermia significantly attenuated the impact of hyperglycaemia on stress fibre formation. Scale bars = 20 μm. NG, normoglycaemia; NT, normothermia; HG, hyperglycaemia; TH, therapeutic hypothermia

**Fig. 6:**
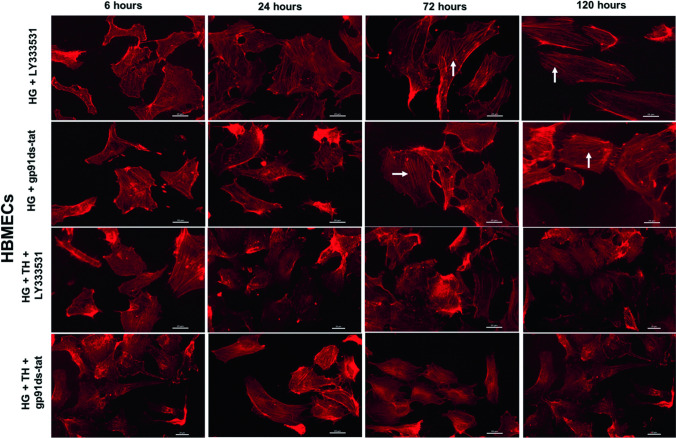
Representative images of actin cytoskeleton staining in human brain microvascular endothelial cells (HBMEC) cultured under hyperglycaemic conditions (6–120 h) in the presence of LY333531 and gp91ds-tat alone or together with therapeutic hypothermia. Albeit treatment with PKC-β or Nox2 inhibitor decreased the extent of stress fibres formation (indicated by white arrows) in HBMECs, the combinatory therapeutic approaches appeared to be more effective in negating the impact of hyperglycaemia. Scale bars = 20 μm. NG, normoglycaemia; NT, normothermia; HG, hyperglycaemia; TH, therapeutic hypothermia

**Fig. 7:**
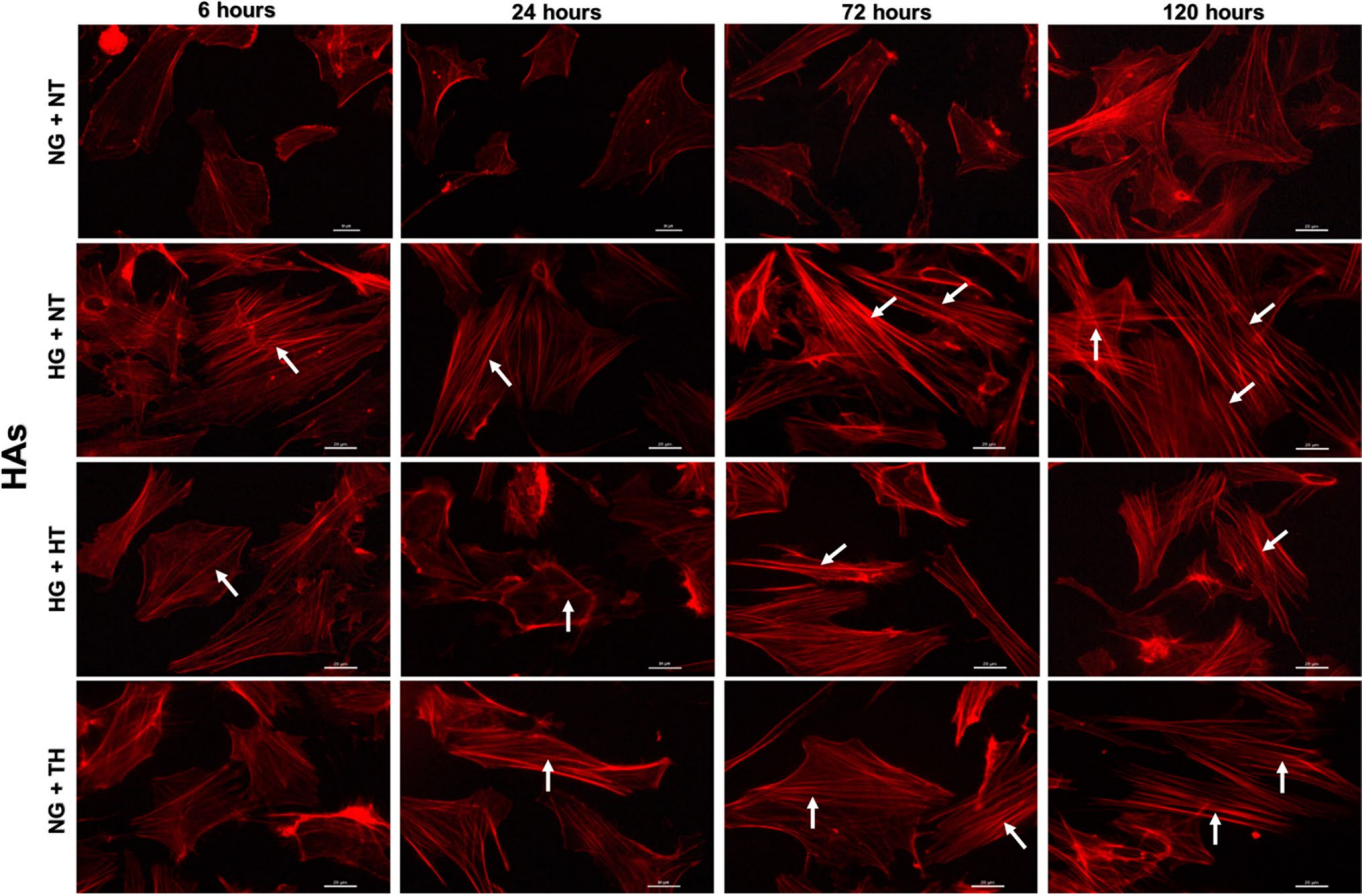
Representative images of actin cytoskeleton staining in human astrocytes (HAs) cultured under normoglycaemic and hyperglycaemic (6–120 h) conditions in the absence or presence of therapeutic hypothermia. Treatment of normoglycaemic HAs with therapeutic hypothermia or exposure to hyperglycaemia markedly elevated actin stress fibre formation (indicated by white arrows) in a time-dependent manner. Therapeutic hypothermia significantly attenuated the impact of hyperglycaemia on stress fibre formation. Scale bars = 20 μm. NG: normoglycaemia. NG, normoglycaemia; NT, normothermia; HG, hyperglycaemia; TH, therapeutic hypothermia

Physical, chemical or humoral changes in the cellular microenvironment dramatically affect actin cytoskeleton which induce a shift from G-actin to F-actin and promote the formation of contractile actin bundles called stress fibres. Assessment of actin cytoskeleton in HBMECs and HAs through rhodamine-phalloidin staining revealed that hyperglycaemia dramatically induced the formation of stress fibres in a time-dependent manner. Exposure to therapeutic hypothermia also promotes stress fibres in both cell lines, maintained in normoglycaemic settings, in a time-dependent manner (Figs. 5–6). While the LY333531, gp91ds-tat and therapeutic hypothermia reduced the amount of hyperglycaemia-induced stress fibres, combination of hypothermia with either inhibitor effectively neutralised the effect of hyperglycaemia on actin cytoskeleton. The quantification of stress fibres intensities further corroborated that combinational therapies were more effective in repairing actin cytoskeleton organisation of HBMECs and HAs compared to any single treatments, as indicated by the lesser magnitude of stress fibres intensities observed in combinational approaches (Fig. 7).

**Fig. 8:**
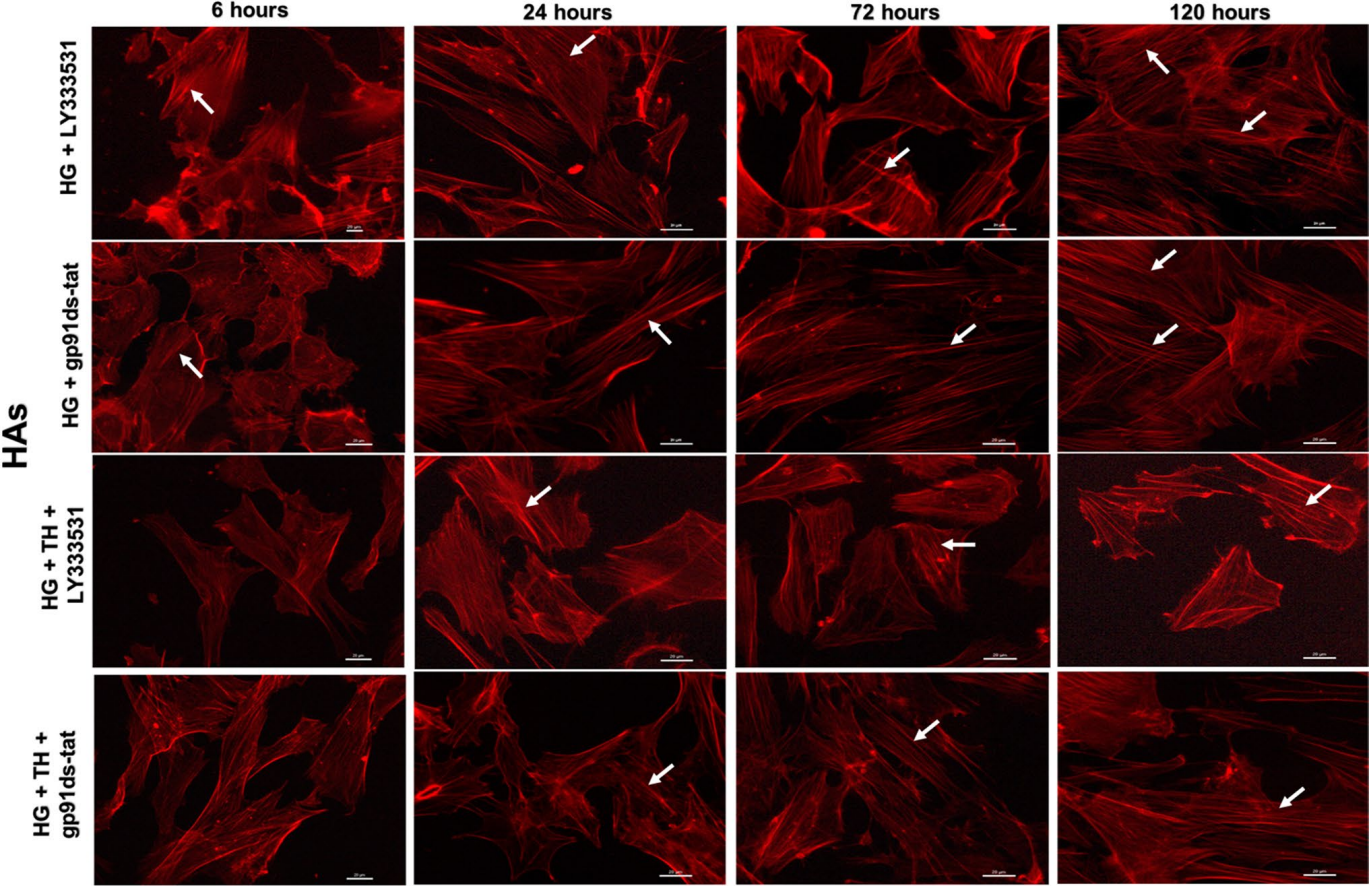
Representative images of actin cytoskeleton staining in human astrocytes (HAs) cultured under hyperglycaemic conditions (6–120 h) in the presence of LY333531 and gp91ds-tat alone or together with therapeutic hypothermia. Although treatment with PKC-β or Nox2 inhibitor alone reduced the extent of stress formation in hyperglycaemic HAs (indicated by white arrows), the magnitude of stress fibre suppression was greater when combined with therapeutic hypothermia. Scale bars = 20 μm. NG, normoglycaemia; NT, normothermia; HG, hyperglycaemia; TH, therapeutic hypothermia

**Fig. 9:**
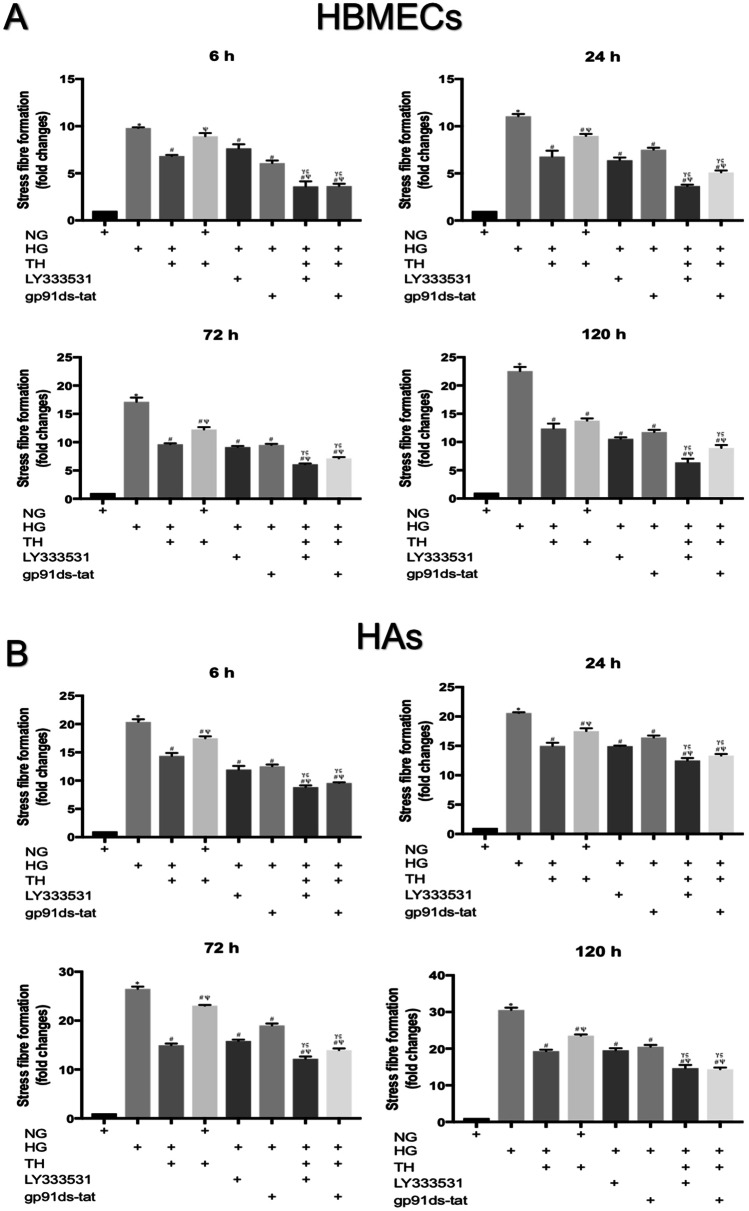
The quantification of the length of stress fibre formation in human brain microvascular endothelial cells (HBMEC) and human astrocytes (HAs) cultured under normoglycaemic and hyperglycaemic (6–120 h) conditions in the presence of therapeutic hypothermia, LY333531, gp91ds-tat or combination of these agents. The length of stress fibres formation in each cell was accumulated and then normalised to cells maintained under normal conditions (NG) to reflect potential changes. Both exposure to hyperglycaemic injury and treatment of normoglycaemic cells with therapeutic hypothermia induced actin stress fibres formation in both cell lines in a time dependent manner. While single therapies with therapeutic hypothermia, PKC-β or Nox2 inhibitor reduced the level of stress fibre formation, the combinatory approaches were more effective in suppressing actin cytoskeleton organisation compared to any single treatment, as evidenced by the emergence of shorter stress fibres in both cells. ^*^P < 0.05 versus normoglycaemia (NG), ^#^*P* < 0.05 versus HG, ^Ψ^*P* < 0.05 versus HG + TH, ^γ^*P* < 0.05 versus HG + LY333531, and ^ς^*P* < 0.05 versus gp91ds-tat. NG, normoglycaemia; HG, hyperglycaemia; TH, therapeutic hypothermia

## Discussion

The BBB, composed of endothelial cells, astrocytic end‐feet, pericytes and basement membrane, serves as an important dynamic semipermeable barrier to maintain cerebral homeostasis. The disruption of BBB, characterised by increased interendothelial cell permeability, is considered as a key pathology in the development of cerebral injury and subsequent neurological impairment during or after an ischaemic stroke (Jiang et al. 2018). The presence of comorbidities, in particular hyperglycaemia, is known to further exacerbate disruption of the BBB after an ischaemic stroke, in that thickening of the capillary basement membrane, induction of oxidative stress and neuronal and endothelial cell apoptosis appear to play an important role (Venkat et al. 2017). Inevitably, ischaemic stroke patients with DM manifest more severe neurological deficits and have higher fatality rates (Li et al. 2013). Besides, diabetic stroke patients treated with rtPA manifest higher rates of intracranial haemorrhage (Masrur et al. 2015; Thorén et al. 2017; Yu et al. 2016). Hence, it is of great therapeutic significance to explore mechanisms that may be utilised to improve outcomes in diabetic stroke patients.

Despite a large number of agents have, over the years, been shown to protect cerebrovascular integrity and function in experimental settings, the subsequent clinical trials performed with the same agents failed to replicate these favourable effects. One of the main drawbacks regarding translational stroke research is that the majority of the agents used in the above-mentioned clinical trials have focused on a single pathway pertaining to either recanalisation of vessels or excitotoxicity to reduce neuronal death, while many other processes are involved spatially and temporally in the pathophysiology of stroke (Alwjwaj et al. 2021; Loach and Bayraktutan 2016). In this context, therapeutic hypothermia capable of targeting multiple pathways, notably oxidative stress, inflammatory responses, metabolic disruption and cell death signals may be a potentially effective procedure (Loach and Bayraktutan 2017). Indeed, therapeutic hypothermia has long been considered as an efficacious therapeutic option for patients with cardiac arrest or for newborns with hypoxic-ischaemic encephalopathy (Bernard et al. 2002; Shankaran et al. 2005). Furthermore, therapeutic hypothermia has been shown to reduce infarct size and neurological deficits by at least 30% in animal models of ischaemic stroke and protect the integrity of the cerebral barrier and its function during ischaemia/reperfusion injury (Mathur and Bayraktutan 2016, 2017; van der Worp et al. 2007). However, whether therapeutic hypothermia may provide cerebrovascular protection in diabetic stroke patients or in the experimental settings of hyperglycaemic cerebral barrier injury remain unclear.

Using an interactive in vitro model of the human cerebral barrier established by concurrent culture of brain microvascular endothelial cells, astrocytes and pericytes, the current study has shown that therapeutic hypothermia can in fact protect BBB from the disruptive effects of hyperglycaemia. In light of the well-established roles of PKC-β and Nox2 in hyperglycaemic BBB damage, studies combining therapeutic hypothermia with the inhibitors of these pathways led to greater protection from hyperglycaemic damage, as shown by the significant increases in TEER and concomitant decreases in NaF flux across the barrier compared to single therapy groups. TEER measurement constitutes the most extensively used methodology to assess BBB integrity. It is based on measurement of the electrical resistance across the monolayers that form the experimental cerebral barrier and indicates the extent of tight junctional unity between neighbouring endothelial cells. Hence, it is considered as a strong and reliable indicator of the tightness of the monolayer (Srinivasan and Kolli 2019; Stone et al. 2019). The measurement of the amount of solute crossing the cellular barrier, on the other hand, provides invaluable information on the extent of openings between tight junctions and therefore serves as an important marker for the functional status of the BBB (Helms et al. 2016; Zucco et al. 2005; Sun et al. 2021). The changes observed in TEER readings and NaF flux in the present study strongly implies a hyperglycaemia-mediated breakdown of the BBB integrity and function, respectively (Helms et al. 2016). Suppression of PKC-β and Nox2 has previously been correlated with decreased apoptosis of endothelial cells exposed to pathological stimuli including hyperglycaemia and inflammatory cytokines like TNF-α (Abdullah and Bayraktutan 2016; Shao and Bayraktutan 2014). It is likely that therapeutic hypothermia may further decrease the endothelial cell apoptosis to improve barrier integrity. Profound decreases observed in oxidative stress, as evidenced by concomitant decreases in pro-oxidant Nox activity and superoxide anion production along with faster and more effective repair of HBMEC and astrocyte damage, attested by wound scratch assay, may also contribute to better responses obtained with the combination therapy.

Polymerisation of actin filaments form linear stress fibres and create tensile centripetal force leading to opening of intercellular gaps between endothelial cells (Chen et al. 2013; Srivastava and Bayraktutan 2017). Experiments focusing on the impact of different therapeutic approaches on actin cytoskeleton have shown better preventive effects with combinatory approach in both HBMECs and HAs. In support of our findings, exposure to therapeutic hypothermia has also been shown to reduce neurological deficits, cerebral infarction, brain oedema volume and BBB hyperpermeability in an experimental model of diabetic stroke in that inhibition of cell death appeared to play a key role (Tu et al. 2019). Intriguingly, despite being highly beneficial in hyperglycaemic settings, therapeutic hypothermia displayed adverse effects on BBB function as well as HBMEC and astrocyte wound closure and actin cytoskeleton organisation under normoglycaemic conditions without affecting the level of oxidative stress. Consistent with these findings, significant decreases in proliferation and increases in apoptosis have been observed in human umbilical vein endothelial cells exposed to therapeutic hypothermia where alterations in the levels of various inflammatory factors, such as IL-6, IL-8, COX-2 and MCP-1 appear to play a role (Diestel et al. 2008). The increases in inflammatory mediators, particularly IL-6, and apoptosis of astrocyte, neuronal and microglial cells after exposing these cells to therapeutic hypothermia in normoglycaemic environment also reported by other studies (Schmitt et al. 2006, 2007), further supporting our findings that therapeutic hypothermia may impair cellular and molecular function of cerebral cells in physiological settings. We also have previously shown that inactivation of PKC-β signalling pathway under normal conditions does not affect actin cytoskeleton formation, RhoA/Rho-kinase/MLC2 pathway activity and ZO-1 expression but increases the expression of a main tight junction protein, occludin in HBMECs (Srivastava et al. 2013). Moreover, the inhibition of NADPH oxidase activity in physiological settings adversely affect endothelial cell growth, an important mechanism in establishment and repair of endothelial barrier (Bayraktutan 2004, 2005).

Exposure to hyperglycaemia induces a series of pathological mechanisms through successive activations of diacylglycerol (DAG) and PKC signalling pathway which in turn activate pro-oxidant enzyme Nox, trigger inflammatory responses and impair cellular architecture (Teodoro et al. 2019). Consistent with the previous studies (Huang et al. 2019; Lorenzi et al. 1985; Zhu et al. 2015), exposure of HBMEC or astrocytes to hyperglycaemia in the current study has also negatively influenced the speed of wound repair of these cells which in part may be explained by excessive release of ROS, notably superoxide anion in these settings. Although superoxide anion plays a crucial role in physiological function, particularly in intracellular communication, neurotransmitter release, and immune defence system, its exaggerated availability in hyperglycaemic settings is coupled to markedly reduce proliferation rate of endothelial cells. In addition to this, the excessive amount of superoxide anion also disrupts normal cellular structure and TJ formation and thus compromises BBB integrity and function (Abdullah et al. 2015; Shao and Bayraktutan 2014; Zanetti et al. 2001). Since Nox is the primary source of superoxide anion and DAG-PKC-Nox pathway is thought to be responsible for much of the above mentioned detrimental effects, it is no surprise that this pathway is known as “dangerous metabolic route in diabetes” and has therefore received a great deal of attention to ameliorate diabetic microvascular and macrovascular complications. Although the inhibition of DAG as the upstream component of this metabolic pathway appears to be the logical option to interrupt this signalling cascade, this has so far not been materialised due to its biochemical complexity (Volpe et al. 2018). Hence, PKC and Nox have emerged as potential therapeutic targets in diabetic stroke and associated complications.

The PKC family of enzymes are classified into three sub-groups based on sequence homology and activation mechanisms, of which PKC-β contributes the most to total PKC activity and plays a seminal role in exacerbating in vitro cerebral barrier damage under hyperglycaemic condition (Shao and Bayraktutan 2013; Srivastava et al. 2013). PKC-β isoform-selective inhibitors have been shown to be safe for human use and exert some degree of therapeutic benefit in patients with diabetic retinopathy (Aiello et al. 2006). They have also been shown to prevent brain oedema by reversing BBB damage in translational settings of diabetic ischaemic stroke (Cipolla et al. 2011). The cerebral barrier-protection realised by PKC-β inhibition may in part be due to the fact that this isoenzyme acts upstream to a wide range of components, notably Nox, known to affect BBB homeostasis. Substantial increases in Nox subunit expression and ensuing overall enzymatic activity have been documented in experimental models of diabetic stroke and correlated with greater cerebral infarct size and severe neurological deficits (Kusaka et al. 2004). Significant increases in Nox activity, particularly Nox2 isoform, and superoxide generation have also been documented in cerebral endothelial cells cultured under hyperglycaemic conditions (Allen and Bayraktutan 2009; Ulker et al. 2004).

Genetic deletion or pharmacological inhibition of PKC-β in HBMECs exposed to hyperglycaemic insult has also been linked to reduced DNA fragmentation rates, caspase 3/7 activity and pro-apoptotic Bax protein release, ascribing a crucial role to PKC-β in endothelial cell apoptosis (Shao and Bayraktutan 2014). In addition, the inhibition of PKC-β has been shown to attenuate increases in endothelial cell urokinase plasminogen activator (uPA) activity evoked by TNF-α, a prominent pro-inflammatory cytokine during the acute phase of an ischaemic injury, implying that PKC-β may also regulate the plasminogen-plasmin system (Abdullah and Bayraktutan 2016). As an upstream regulator of NF-κB, a transcription factor that regulates multiple aspects of innate and adaptive immune reactions, the increased availability of PKC-β in diabetic animals has been shown to account for vascular inflammatory response and accelerated atherosclerosis (Kong et al. 2013; Lutzny et al. 2013). Indeed, acquisition of better integrity and function of BBB in an animal model of acute inflammation treated with a PKC-β inhibitor further emphasise the seminal role of PKC-β in induction of inflammatory responses and ensuing BBB dysfunction (Lanz et al. 2013). It is of note here that PKC-β-mediated phosphorylation and subsequent degradation of occludin and ZO-1, major tight junction proteins, may also be involved in BBB dysfunction (Avila-Flores et al. 2001; Chen et al. 2002; Murakami et al. 2012).

Considering the diversity of pathways targeted by PKC-β and the consistent association of Nox with these pathways, the present study specifically investigated the preventive/restorative impact of Nox2 inhibition alone or with therapeutic hypothermia on BBB in hyperglycaemic settings. Data generated show better barrier-protective impact when therapeutic hypothermia is applied along with Nox2 inhibitor. The mechanism of reparative effects of these combinational therapies is illustrated in Fig. 9. The discovery of remarkably smaller cerebral infarcts size in Nox2-deficient mice subjected to MCAO and considerably lower oxidative stress level after knockdown of Nox-2 activity in neuronal cells exposed to hyperglycaemia with ischaemic damage corroborate the therapeutic benefits of Nox2 inhibition during hyperglycaemic injury (Kahles et al. 2007; Zeng et al. 2018) Figs. 8–10.

**Fig. 10:**
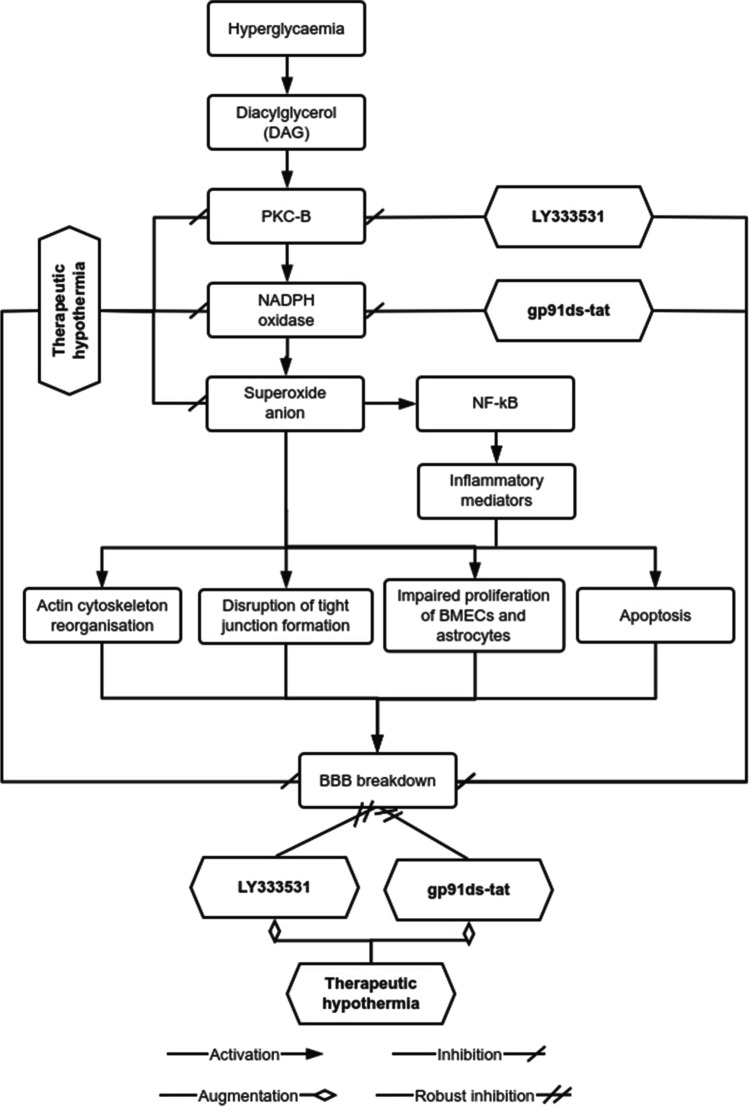
The effect of hyperglycaemia on blood–brain barrier (BBB) integrity in the absence or presence of therapeutic hypothermia alone or together with inhibitors targeting PKC-β or Nox2. Through successive activation of diacylglycerol and PKC-β, hyperglycaemia increases the activity of NADPH oxidase which ultimately leads to BBB breakdown through generating excessive levels of superoxide anion, activating transcription factor NF-κB and regulating the synthesis, release and recruitment of various inflammatory mediators. Induction of stress fibre formation, tight junction reorganisation, apoptosis as well as inhibition of the proliferative and migratory capacity of endothelial cells and astrocytes also appear to contribute to the overall BBB damage. While treatments with therapeutic hypothermia alone effectively suppress the deleterious effects of hyperglycaemia on BBB, its combination with approaches negating the activity of PKC-β or Nox2 provide greater BBB-restorative capacity

Accumulating evidence has displayed that the combination of therapeutic hypothermia with other therapeutic agents achieves better effectiveness in repairing neurovascular damage following ischaemic stroke. For instance, the combination of therapeutic hypothermia with calcium and glutamate antagonist (magnesium) and with an antioxidant (tirilazad) enhances the restorative role of therapeutic hypothermia on cerebrovascular damage after ischaemia, as ascertained by marked reductions infarct volume and neurological deficits in MCAO rats (Zausinger et al. 2003). Similarly, the combination of mild hypothermia with dizocilpine (glutamate antagonist), human urinary kallidinogenase (kinin activator) or neuroglobin (neuron oxygen-binding protein) is more efficacious in protecting ischaemia/reperfusion-induced neuronal injury compared to single treatment regimens (Gao et al. 2014; Wei et al. 2018). Better protection of the cerebral barrier integrity and function through combination of a PKC-β or a Nox2 inhibitor with therapeutic hypothermia in the present study further corroborate the higher efficacy of combinational strategy in hyperglycaemic conditions.

In conclusion, the current study has shown that therapeutic hypothermia augments the BBB-restorative effects of PKC-β or Nox2 inhibition in hyperglycaemic settings by reducing oxidative stress, restoring actin cytoskeleton formation and increasing the proliferation and directed migration of HBMECs and HAs, two main cellular components of the BBB.

## Data Availability

All data generated or analysed during this study are included in this published article.
